# Air Pollution and Effects of Tropospheric Ozone (O_3_) on Public Health

**DOI:** 10.3390/ijerph22050709

**Published:** 2025-04-30

**Authors:** Pavlos Vongelis, Nikolaos G. Koulouris, Petros Bakakos, Nikoletta Rovina

**Affiliations:** Respiratory Function Laboratory, 1st Department of Respiratory Medicine, Sotiria Hospital, Medical School, National and Kapodistrian University of Athens, GR-11527 Athens, Greece; koulnik@med.uoa.gr (N.G.K.); pbakakos@med.uoa.gr (P.B.); nikrovina@med.uoa.gr (N.R.)

**Keywords:** chronic airway narrowing, tropospheric ozone (O_3_), sulfur dioxide (SO_2_), particulate matter (PM_10_), asthma, COPD, nitrogen oxides (NO_x_)

## Abstract

Air pollution is a significant and widespread issue that presents serious challenges for both human health and the environment because of the presence of a variety of harmful substances in the air, such as tropospheric ozone (O_3_), particulate matter (PM_10_), nitrogen oxides (NOx), sulfur dioxide (SO_2_), and carbon monoxide (CO). In this research, the aim is to evaluate the current evidence for the harmful effects of air pollution on human health, focusing on tropospheric ozone, and to highlight the need for further research in the future. The objective is to evaluate recent data on the respiratory and cardiovascular risks caused by air pollution, the potential association between climate change due to air pollution and human disorders, and the subsequent economic burden. A systematic search of the literature is conducted using PubMed, Scopus, Web of Science, and regulatory reports (EPA), focusing on peer-reviewed studies, epidemiological analyses, and clinical and experimental studies. The key findings indicate that O_3_ exposure contributes to inflammatory lung injury and to the worsening of preexisting conditions like asthma and COPD, is associated with cancer, and also has numerous negative impacts on neurological, metabolic, and reproductive health, combined with increased healthcare costs. These findings highlight the significance of O_3_ pollution as a major public health concern, emphasizing the need for immediate measures to decrease emissions and effective policies to protect the climate and the health of the individuals.

## 1. Introduction

Exposure to an air pollutant is defined as any contact of that pollutant with a surface of the human body, either external, as in the case of the skin, or internal, as in the case of the respiratory epithelium [1,2]. Air pollution is widely acknowledged by the scientific community as a significant threat to human health [3], with its effects varying across regions. Notably, exposure levels in Eastern Europe are lower compared to the rest of Europe [4,5]. The issue of human exposure to air pollution and its impact on public health is an intricate and complex matter that involves the interplay of multiple factors, and as a consequence, it is quite difficult to measure them accurately.

Many studies in almost all regions around the world have shown an association between daily increases in pollution levels and various human health effects, such as respiratory diseases [6,7], asthma [8], COPD [9], cardiovascular diseases [10], cerebrovascular diseases and strokes [11], among others. Various theories have been put forward on the etiology of these effects of environmental pollution [12], but in general, this area is still poorly understood, and no consensus has been reached by the scientific community on which components of air pollution [13] are the most harmful [14]. Evidence also exists that air pollution affects multiple organs through these processes, including the development or exacerbation of chronic diseases such as cancer, cardiovascular, cerebrovascular, and metabolic diseases [15]. The World Health Organization (WHO) estimates that 12.6 million deaths per year worldwide are caused by environmental factors [16], and studies also estimate the impact of particulate matter as being a factor in 9 million premature deaths [17].

Oxidative stress is caused by an imbalance between the production and accumulation of oxygen reactive species (ROS) in cells and tissues and the ability of a biological system to detoxify these reactive products with antioxidants. ROS are normally generated as by-products of oxygen metabolism; despite this, environmental stressors (i.e., UV, ionizing radiations, pollutants, and heavy metals) and xenobiotics (i.e., antiblastic drugs) contribute to greatly increasing ROS production, therefore causing the imbalance that leads to oxidative stress. Particularly for the respiratory system, when lung epithelial cells are exposed to particulate matter (PM), which is a combination of metals and other secondary substances found in air pollution, it triggers the release of reactive oxygen species, leading to inflammation, cell death, and organ damage [18,19,20,21]. In addition, epigenetic changes, such as somatic mutations and DNA methylation caused by environmental exposure, can influence the development of chronic diseases and cancer [22,23]. In the respiratory system, immune cell interactions, changes in the pulmonary microbiota, and viral activation also contribute to increased susceptibility to pneumonia as well as the exacerbation of respiratory diseases [15].

The main air pollutants—for which there is accumulated evidence of adverse health effects—include particulate matter (PM), nitrogen oxides (NOx and NO_2_), ozone (O_3_), sulfur dioxide (SO_2_), and carbon monoxide (CO) [24]. The levels of these pollutants are continuously monitored around the world to determine the air quality in a given area.

Tropospheric ozone (O_3_) presents low water solubility. Due to that, it is not effectively removed by the upper respiratory tract and has the ability to penetrate deeply into the lungs [1,25]. Being a highly reactive gaseous pollutant, tropospheric ozone (O_3_) exerts inflammatory effects on the respiratory system. Oxidative stress and airway inflammation are caused more often in allergic subjects by oxygen radicals [1,26]. Moreover, short-term exposure to O_3_ increases both the risk of current asthma as well as that of an asthma exacerbation [1,27,28]. It has been estimated that 9–23 million annual asthma emergency room visits globally in 2015 may be attributed to tropospheric ozone (O_3_), and that represents 8–20% of the annual number of global hospital visits [1,29,30].

According to experts, climate change will have a significant impact on future tropospheric ozone (O_3_) levels, causing concentrations to rise in many regions despite the implementation of emission control measures. This will result in a change in weather patterns, extending the period of elevated tropospheric ozone concentration occurrence, which in turn will lead to an increase in the duration, frequency, and severity of ozone episodes [31].

In the time of climate change and with its deleterious effects across multiple levels, there is a continuous need for raising awareness of these effects. Since O_3_ exposure contributes to inflammatory lung injury and to the worsening of pre-existing respiratory conditions like asthma and COPD, is associated with increased healthcare costs, and poses a major public health concern, this review aims to sum up and discuss the mechanisms through which air pollutants and especially tropospheric ozone can enter the respiratory system and lead to harmful effects.

## 2. Materials and Methods

**Figure 1:**
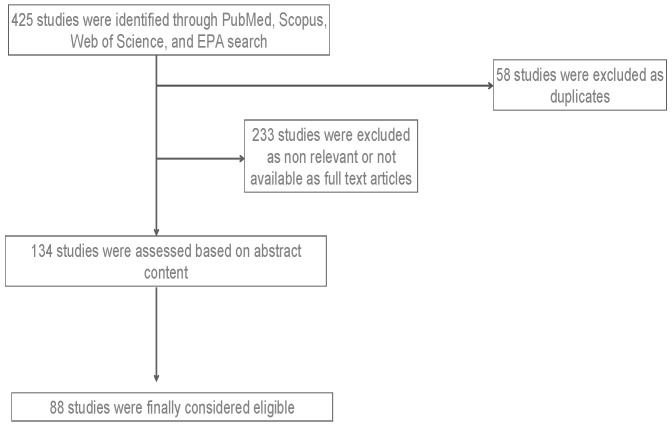
PRISMA flowchart for the literature screening.

We undertook a systematic review of the literature following the Preferred Reporting Items for Systematic Reviews and Meta-Analyses (PRISMA) statement [24]. The literature search was conducted across PubMed, Scopus, Web of Science, and regulatory reports (EPA) to identify studies on the health effects of air pollution, focusing on tropospheric ozone (O_3_). Search terms included “air pollution,” “ozone exposure,”, “tropospheric ozone AND health effects”, “respiratory health,” and “cardiovascular effects.” The search was restricted to the period from 1 January 2015 to 31 October 2024 and to publications in the English language for which an abstract was available. We searched for original studies (randomized clinical trials [RCTs], nonrandomized trials, post hoc analyses of RCTs, and observational studies). We included studies with qualitative data related to the impact of air pollution and tropospheric ozone on health, and specifically on the respiratory system. Studies focusing on indoor pollution and natural disasters were excluded. Unpublished studies, duplicates, or studies without an abstract were also excluded. A total of 425 studies were identified and reviewed, 134 studies were considered initially eligible based on the title and abstract, and 88 were finally included in the data synthesis (Figure 1).

## 3. Air Pollution and Respiratory System

It is widely known that air pollutants have several adverse effects on human health. Outdoor air pollutants, driven mostly by human activities such as work related to maritime, industrial facilities, traffic on the roads, and natural disasters, operate as human hazards. Chemically, there are particular molecules which are mostly detected in the environment: ozone (O_3_), carbon monoxide (CO), nitrogen dioxide (NO_2_), sulfur dioxide (SO_2_), and other forms of organic pollutants [4]. However, particulate matter, meaning the mixture of airborne particles from natural and human activities [32,33] which vary in size and composition, also demonstrate an important role in airway dysfunction. Since most pollutants enter the body through the airways, the respiratory system is the first to be affected by diseases caused by air pollution. The extent of damage depends on the amount of inhaled pollutants and their deposition in target cells [34].

The respiratory system stands out as the system that encounters air pollution to a greater degree than any other human system. Daily, 10 cubic meters of atmospheric air of different temperatures and different levels of particulate matter and humidity are inhaled [35,36,37,38,39]. Accordingly, persistent efforts are being made by scientists to address the harmful effects of air pollution on human health by controlling the sources of air pollution [38,39,40,41,42,43].

## 4. Tropospheric Ozone and Health Effects

Surface ozone is a secondary pollutant formed in the presence of ultraviolet radiation from the sun and formed predominantly by a series of photochemical reactions of some volatile organic compounds (VOCs) and nitrogen oxides (NO_x_) [44].

The majority of surface tropospheric ozone, around ninety percent, is produced through photochemical reactions within the troposphere itself, while the remaining ten percent comes from ozone entering the troposphere from the stratosphere above [44,45,46].

**Figure 2:**
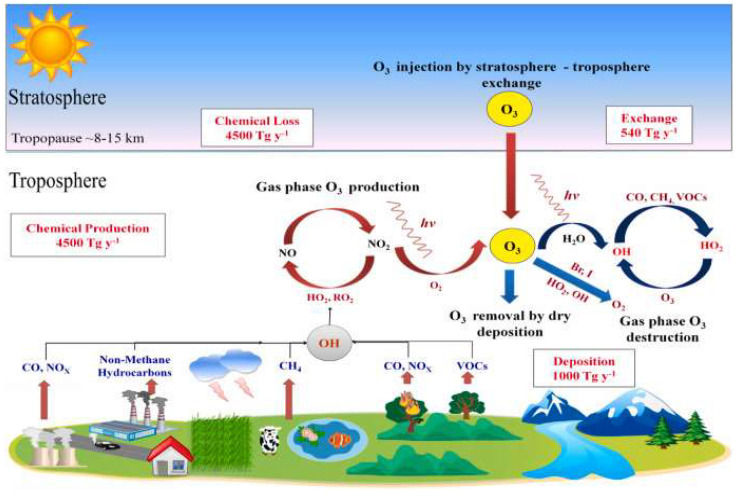
Formation of tropospheric ozone [53] (available from: https://doi.org/10.1016/j.envres.2023.116816).

A particularly important role in the dispersion of global tropospheric ozone levels and in the transport of the photochemical cloud itself and its precursors is played by the synoptic atmospheric circulation systems [45,47,48,49]. Briefly, the main production of ozone (O_3_) is a result of the oxidation of methane (CH_4_) and non-methane hydrocarbons (NMHCs) when nitrogen oxides are present (NO_x_) [50]. The stratosphere also contains an amount of ozone, primarily produced by UV radiation [51]. Some of this ozone enters the troposphere through tropopause folds, jet streams, and deep convection, contributing an estimated 340–930 Tg (teragrams) per year [51]. There are several systems based on these facts which explain the circulation of tropospheric ozone in the atmosphere. For example, Brewer–Dobson circulation is a large-scale atmospheric pattern in which air from the tropical troposphere ascends into the stratosphere, then gradually moves toward the poles before descending back down, and plays a key role in transporting stratospheric ozone from the tropics to higher latitudes [52], where polar vortices and strong, persistent low-pressure systems over the poles, especially in winter, can isolate ozone-rich or ozone-poor air masses, influencing seasonal depletion. Thus, when the vortex weakens in spring, ozone from higher altitudes or mid-latitudes can mix into the region [52]. A schematic representation of the origin of tropospheric ozone is shown in Figure 2.

The annual production rate of tropospheric ozone using the ozone deposition method is 422 ± 210 Tg/year [53,54,55]. The input of ozone from the stratosphere is determined to be 552 ± 168 Tg/year and the dry deposition to the ground is about 1003 ± 200 Tg/year (where teragrams O_3_/year equals 10^15^ g/year). The concentrations of nitrogen oxides and hydrocarbons and the intensity of solar UV radiation play a particularly crucial role in the extent of tropospheric ozone formation [54,55,56,57].

Tropospheric ozone is a highly irritating gas, with a characteristic strong odor and a strong oxidizing effect on the airways. Airway hyperresponsiveness as well as inflammation are induced by acute human exposure to tropospheric ozone (O_3_). As expected, it has also been proposed as a possible explanation for the effect of air pollutants on asthmatic individuals [55,56,57,58].

Tropospheric ozone reacting primarily with the antioxidant elements of the overlying fluid in the respiratory epithelium leads to antioxidant depletion but also to a basic level of imbalance between antioxidants and non-antioxidants in the cells and in the extracellular fluid, which essentially defines oxidative stress. The above process contributes to both oxidation and structural changes in the reactive products of proteins and lipids as well as molecules. Horstman et al. concluded that short-term exposure to tropospheric ozone levels of less than one hundred and twenty micrograms per cubic meter of air for a period of seven hours causes significant damage to the function of the pulmonary system [59,60].

The seriousness of the clinical presentation and the manifestation of symptoms are primarily influenced by the levels of tropospheric ozone, the length of time a person is exposed to it, and the rate at which they breathe [59,60,61]. Respiratory rate is highly correlated to how physically active an individual is.

Horstman et al. documented the substantial differences in the responses of normal individuals after exposure to tropospheric ozone [59,60,61,62]. After exposure to 240 micrograms per cubic meter of air for about seven hours, the drop in FEV_1_ values ranged up to 37%. On the other hand, the individual responses remained constant at 160–200 micrograms per cubic meter of air, respectively [59,60,61,62,63].

After the exposure of normal subjects to high concentrations of tropospheric ozone, an increase in airway reactivity was found. This increase in airway reactivity was associated with an increase in the proportion of neutrophils recovered during the bronchoalveolar lavage, leading to the conclusion that tropospheric ozone is responsible for the acute neutrophilic airway inflammatory response recorded 18 h after exposure [1,2,60,61]. However, there are extremely large individual variations in the response to different tropospheric ozone concentrations. The response to high tropospheric ozone levels is influenced by polymorphisms in the genes coding for oxidative defense mechanisms and by pre-existing inflammatory airway diseases such as asthma and co-exposure to allergens [60,61,62,63,64]. As a consequence, the impact of tropospheric ozone extends beyond the general healthy population to include those who suffer from Chronic Obstructive Pulmonary Disease (COPD), bronchial asthma, and chronic airway narrowing [31].

Studies have also demonstrated that tropospheric ozone (O_3_) can, at ambient concentrations, induce an airway reaction in sensitive individuals during both heavy and strenuous exercise and moderate exercise. In addition, the effects of sulfur dioxide (SO_2_) and various inhaled allergens are associated with ozone effects in asthmatic patients, while no association has been recorded in non-asthmatics [2,34,64].

**Figure 3:**
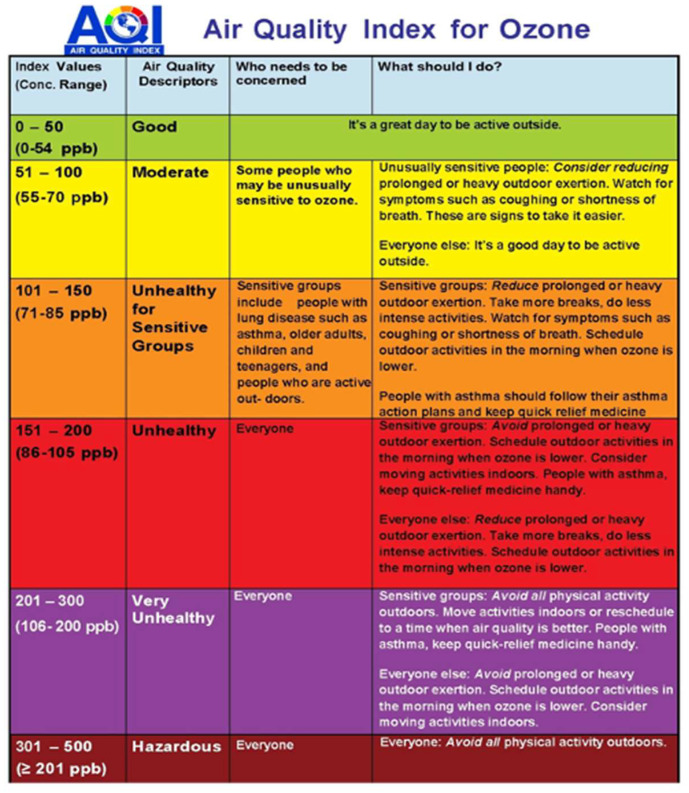
Limit values for tropospheric ozone (O_3_) [68] (available from: https://www.mdeq.ms.gov/air/other-air-information/understanding-ozone-and-the-aqi/ accessed on 2 September 2023).

Tropospheric ozone, apart from inducing an influx of neutrophils into the alveolar spaces and increasing bronchial reactivity, as already mentioned above, also increases the viscosity and quantity of mucus in the tracheobronchial tree and the transmissibility through the airway epithelium. The bronchial epithelium exhibits an elevated permeability, which allows for the absorption of harmful air pollutants and facilitates the presence of inflammatory cells on the surfaces of the airways. Neutrophils are the main source of production of neutrophilic elastase, ultimately leading to the development of emphysema [34,65,66,67]. In addition, the oxidation of polyunsaturated fatty acids in the cell membrane is induced by increased tropospheric ozone levels. However, at the cellular level, the precise and detailed mechanism through which tropospheric ozone acts is currently under investigation [60,61,62,63,66]. The limit values for tropospheric ozone (O_3_) are shown in Figure 3.

## 5. The Relationship Between Tropospheric Ozone and the Climate

Tropospheric ozone is one of the greenhouse gases that has a direct effect on global warming. In contrast to other greenhouse gases, the residence time of tropospheric ozone in the lower levels of the atmosphere does not become sufficient for its homogeneous dispersion, resulting in a geographical limitation of its effect to be near its production area [44,45,46,47,48]. The potential consequences of the indirect interaction between tropospheric ozone and the climate may have a considerable impact. Increases in ozone concentrations are associated with an increase in the number of heatwave days in a year as well as more frequent occurrences of extreme weather events [45,46,47,48,49,69]. As a result of elevated surface temperatures, trees exhibit an increased emission of volatile organic compounds, thereby contributing to the augmentation of ozone levels. The degradation of trees and forests, primarily caused by wildfires, results in the substantial accumulation of carbon dioxide in the atmosphere (CO_2_) and the heightened incidence of the ozone haze phenomenon, and these processes culminate in the acceleration of global warming [44,45,46,47,48]. Consequently, the indirect association between tropospheric ozone and the climate is becoming more apparent with the elapsing of time, creating concerns for the future [53,54,55].

These interactions between tropospheric ozone and the climate are causing direct and indirect adverse effects on human health [14]. The direct effects involve the adverse effects that the inhalation of tropospheric ozone induced human. The indirect effects are more complex. Studies have shown that the exposure to higher temperatures leads to increased cardiovascular (3.44%, 95% CI 3.10–3.78), respiratory (3.60%, 3.18–4.02), and cerebrovascular (1.40%, 0.06–2.75) mortality [70]. Thus, the extreme heatwave effects and the increase of the world’s temperature are caused to a great extent by the accumulation of tropospheric ozone, which can lead to serious adverse effects on public health.

## 6. Absorption of Ozone—Similarities and Differences with Particulate Matter

The lungs play a critical role in absorbing both particulate matter and gases from the environment. In recent years, a number of experimental studies have been conducted to investigate tropospheric ozone using a combination of radioactive and stable isotopes [71,72,73,74].

As has been reported, when tropospheric ozone comes into contact with the lining of the respiratory system, it causes a reaction with the liquid components present. Tropospheric ozone molecules are unable to pass through the thin layer of liquid lining in the lungs, particularly when its thickness is more than 10^−1^ μm, without undergoing a reaction with the components of the lining. The thickness of the liquid lining layer varies throughout different parts of the lungs. In the upper airways, it ranges from 15 to 20 μm, whereas in the alveoli, it ranges from 10^−1^ to 10^−2^ μm [68,69,70,72,73,74,75]. This variation in thickness is primarily attributed to differences in the thickness of the liquid subphase, as well as of the mucus layer [32,66,73,74,75].

The pattern of breath has a significant impact on the absorption of atmospheric gases by the respiratory tree. When we breathe through our nose, particulate matter is exposed to a larger absorption surface area, primarily in the nose and the nasopharynx region. As a result, the number of particles reaching the lungs is significantly lower compared to when we breathe through our mouth [35,65,66,67]. This is particularly crucial because during intense physical activity there is a noticeable increase in the amount of gas that reaches the lungs due to individuals breathing through the mouth. The respiratory tract removes most of the inhaled air pollutants and 90% of tropospheric ozone is extracted from the entire system. In contrast, sulfur dioxide (SO_2_) uptake in humans occurs almost completely in the nose, upper airways, and nasopharynx [34,35,71,75]. Accordingly, only small to minimal amounts of sulfur dioxide actually make it to the point of lung gas exchange. The concentration of the gas inhaled plays a significant role in determining the percentage of absorption through the nose. For instance, when the concentration of inhaled gas is low, the nasal absorption reduces to about 5%. In contrast, tropospheric ozone is not removed effectively from the nasal cavity and up to 40% of the inhaled gas is absorbed [35,71,76,77,78].

In an attempt to evaluate the absorption of tropospheric ozone, various anatomical models have been used that incorporate the branching patterns of the air ducts with the intake patterns of reactive gases [35,75,76,77,78,79]. Uptake is quantified by measuring the amount of gas absorbed per unit area, per minute, and per microgram of tropospheric ozone present in the surrounding air [76,77,78,79].

In regard to tropospheric ozone, the highest absorption occurs in the tracheal region, but then gradually decreases as it moves towards the terminal bronchioles. When it approaches the alveoli and alveolar pores, the amount of ozone in the tracheal region is low [74,75,76]. In the alveoli, the amount of tropospheric ozone absorbed by the tissue is almost the same as the amount of ozone that reaches the surface due to the thin layer of liquid lining [32,75,76,77,78,79].

Experts have extended the modeling of tropospheric ozone uptake patterns according to the human lungs of both children and adults. Physical activity has a significant impact on the amount of ozone that is taken in by the distal airway [71,74,77].

## 7. Discussion

The effects of air pollution and especially ozone are of high interest for humankind. Short-term exposure to increased levels of air pollution has the potential to worsen respiratory symptoms in both healthy individuals and those with pre-existing lung conditions such as asthma and COPD. Several studies, particularly in urban and industrialized regions, have indicated the potential role of pollutants in the pathogenesis of asthma and COPD [80]. Exposure to ambient O_3_ and household air pollution might be important risk factors for COPD among young adults [81]. On high ozone days, increased school absences, increased visits to emergency rooms, and increased hospital admissions are reported [82,83,84,85], while long-term exposures to ozone have been associated with lower lung function and deteriorated or abnormal lung development in children [86,87]. Recent data suggest a potential causal relationship between long-term exposure to high-level ambient O_3_ and increased risks of adult-onset asthma [88].

In both the WHO guideline and the EPA ozone standard, more susceptible populations are considered. The greatest effects of tropospheric ozone are mostly observed in the elderly, children, athletes, and people that work outdoors in general (i.e., constructors, fire-fighters). The combined effects of tropospheric ozone and other air pollutants have a more significant negative impact on the health of women, also leading to potential harm to developing fetuses. Apart from these groups, vulnerable people include individuals with obesity, diabetes mellitus, pneumonia, cystic fibrosis (CF), and cardiorespiratory diseases [89].

Besides airway obstructive diseases, other respiratory pathologies affected by airborne pollutants include lung cancer, respiratory infections, and lung fibrosis [90]. Indeed, there are accumulating data documenting that various ambient air pollutants can induce and exacerbate pulmonary fibrotic processes. Notably, an increased correlation between prolonged exposure to elevated levels of O_3_ and NO_2_ and an increased risk of acute exacerbations in patients with idiopathic pulmonary fibrosis has been shown in epidemiological studies [91].

Urban populations are subjected to more deleterious effects of pollutants than populations in rural areas. In urban areas, more pollutants are commonly found in combination, such as O_3,_ particulate matter, diesel exhaust particles, NO_2_, SO_2_, and black carbon. Furthermore, synergistic interactions between O_3_ and other air pollutants can significantly enhance lung inflammation and damage, leading to exacerbations of chronic lung diseases, and posing a greater risk to respiratory health than individual pollutants alone. The synergistic effects of co-exposure to O_3_ and PM_2.5_ have been shown in animal studies where amplified lung inflammatory responses were documented. Furthermore, the synergistic effect of O_3_ and diesel exhaust particles on the lungs, exacerbating symptoms and chronic lung diseases, has also been shown [92,93,94]. The simultaneous inhalation of O_3_ and carbon black particles leads to heightened lung inflammation and functional decline, oxidative stress, epithelial injury, and subsequent inflammatory responses [95]. The co-existence of NO_2_ and O_3_ increases the sensitivity of the airways to allergens and other irritants [96]. When combined with O_3_, SO_2_ induces airway responsiveness and inflammation, leading to more severe respiratory symptoms [96]. As a consequence of ambient pollutant exposure, patients with chronic respiratory diseases in urban areas present with increased respiratory symptoms and more hospital admissions. Daily air pollution exposure is positively associated with an increase in short-acting beta-agonist (SABA) use in patients with asthma and COPD [88]. Exposure to O_3_, NO_2_, and SO_2_ significantly impacts COPD hospitalization caused by air pollution [97]. Recent studies suggest that increased long-term exposure to air pollutants may be linked to the worsening of interstitial lung diseases, an increased risk of acute exacerbations [98], a worse quality of life, and poorer lung function [99].

The consequence of the effect of air pollution and tropospheric ozone is manifested as a decline in people’s overall well-being, reduced earning potential and work productivity, higher rates of illness, increased morbidity and mortality, premature death, and increased expenses related to healthcare and hospitalization. A clear illustration of the economic impact of this effect is the state of California, where it was estimated that from 2005 to 2007 the total hospital costs of USD 193 million and USD 58 million were attributed to air pollution caused by tropospheric ozone [100]. These costs were in turn a heavy burden both for the patients themselves and for the national health systems. Ozone concentrations are expected to increase in the following years, especially in urban areas. Apart from climate change and temperatures being mainly elevated, the sun radiation and particularly emissions from ozone precursors such as nitrogen oxides and volatile organic compounds are the causes of ozone formation with all the accompanying negative impacts on human health. Accordingly, the reduction in exposure to ozone and other pollutants caused by human interventions/sources is of major importance. Experts estimate that in the next 30 years, medical costs due to increased ozone concentrations and other air pollutants will approach USD 580 million and mortality will exceed 2 million patients [101]. According to a European study, out of the total of EUR 370 billion in commercial losses in 2005, a significant portion of EUR 220 million was attributed to elevated levels of tropospheric ozone [102].

There are certain limitations of this review. Only publications in English were included from the databases. The harmful effects of ozone on human respiratory health may be difficult to determine accurately due to the combined effect of other pollutants and the pre-existence of respiratory diseases such as asthma and COPD. The use of particular search items may present a limitation of this review, since not all possible aspects of this broad topic may have been addressed. For example, terms related to vulnerable populations, differences between rural and urban areas, and pollutant interactions were not sought out and analyzed in depth. Finally, the heterogeneity of the studies and their design, the differences between geographical areas, and the interactions between pollutants are confounders that may compromise the validity and generalization of this review.

## 8. Conclusions

The findings of this review have attempted to provide an understanding of the complex mechanisms through which air pollutants can enter the respiratory system, leading to oxidative stress that harms human health and worsens underlying respiratory health conditions. The diverse responses that individuals demonstrate against air pollution, which can be influenced by factors such as epigenetic predisposition and underlying health conditions, are an important area that requires further examination. In this regard, a deeper understanding of the complex mechanisms that lead to an increased susceptibility of vulnerable populations to air pollution is needed. Climate change and related environmental factors such as ozone affect the respiratory system by increasing the prevalence, severity, and healthcare burden of respiratory diseases. It is obvious that the most efficacious measure to prevent the onset and deterioration of chronic respiratory diseases is reducing airborne pollution and extremely high levels of O_3_. Multidisciplinary boards should collaborate on the implementation of comprehensive strategies to curb pollution, including regulatory measures, technological innovations, and public awareness campaigns to alleviate the burden of these debilitating conditions on global health. Special awareness is needed for pollutant effects on global health in specific regions and vulnerable populations.

Finally, further research based on homogenously designed studies and a better understanding of the complex pathways underlying chronic inflammatory lung diseases should be the goal in the pursuit of novel and/or additional therapeutic targets for intervention and for the implementation of personalized healthcare, especially for individuals at risk of the adverse effects of air pollution.
